# Consensus on context-specific strategies for reducing the stigma of human immunodeficiency virus/acquired immunodeficiency syndrome in Zambézia Province, Mozambique

**DOI:** 10.1080/17290376.2014.885847

**Published:** 2014-02-17

**Authors:** Abraham Mukolo, Isabel Torres, Ruth M. Bechtel, Mohsin Sidat, Alfredo E. Vergara

**Affiliations:** a MSc, MPH, PhD, Clinical Assistant Professor, is affiliated to the Vanderbilt Institute for Global Health, Vanderbilt University, Nashville, TN, USA; b BA, Technical Officer at Family Health International, Maputo, Mozambique; c MSc, Country Director, VillageReach, Maputo, Mozambique; d MD, MSc, PhD, Chair, Department of Community Health, Faculty of Medicine, University Eduardo Mondlane, Maputo, Mozambique; e PhD, Epidemiologist, Centers for Disease Control and Prevention, Maputo, Mozambique. Affiliated to Vanderbilt Institute for Global Health, Nashville, TN, USA

**Keywords:** stigma reduction, consensus, HIV/AIDS, Mozambique, réduction de la stigmatisation, consensus, le VIH/SIDA, Mozambique

## Abstract

Stigma has been implicated in poor outcomes of human immunodeficiency virus (HIV)/acquired immunodeficiency syndrome (AIDS) care. Reducing stigma is important for HIV prevention and long-term treatment success. Although stigma reduction interventions are conducted in Mozambique, little is known about the current nature of stigma and the efficacy and effectiveness of stigma reduction initiatives. We describe action research to generate consensus on critical characteristics of HIV stigma and anti-stigma interventions in Zambézia Province, Mozambique. Qualitative data gathering methods, including indepth key-informant interviews, community interviews and consensus group sessions, were utilized. Delphi methods and the strategic options development analysis technique were used to synthesize qualitative data. Key findings are that stigma enacted by the general public might be declining in tandem with the HIV/AIDS epidemic in Mozambique, but there is likely excessive residual fear of HIV disease and community attitudes that sustain high levels of perceived stigma. HIV-positive women accessing maternal and child health services appear to shoulder a disproportionate burden of stigma. Unintentional biases among healthcare providers are currently the critical frontier of stigmatization, but there are few interventions designed to address them. Culturally sensitive psychotherapies are needed to address psychological distress associated with internalized stigma and these interventions should complement current supports for voluntary counseling and testing. While advantageous for defining stakeholder priorities for stigma reduction efforts, confirmatory quantitative studies of these consensus positions are needed before the launch of specific interventions.

## Résumé

La stigmatisation a été impliquée dans de piètres résultats des soins VIH/SIDA. Réduire la stigmatisation est important pour la prévention du VIH et le succès thérapeutique à long terme. Bien que les interventions visant à réduire la stigmatisation sont menées au Mozambique, on en sait peu sur la nature réelle de la stigmatisation et de l'efficacité des initiatives visant à réduire la stigmatisation. Nous décrivons la recherche-action afin de générer un consensus sur les caractéristiques essentielles de la stigmatisation et des interventions contre la stigmatisation dans la province du Zambèze, au Mozambique. Les méthodes qualitatives de collecte de données, y compris des entretiens en profondeur avec des informateurs clés, les interviews communautaires et des séances de groupe de consensus, ont été utilisées. Méthodes Delphi et la technique de l'analyse des options stratégiques de développement (SODA) ont été utilisées dans la synthèse de données qualitatives. Les principales conclusions sont que la stigmatisation promulguée par le public a diminué en tandem avec l'épidémie de VIH/SIDA au Mozambique, mais il est probable que la peur résiduelle excessive de l'infection à VIH et des attitudes de la communauté persiste. Les femmes séropositives qui ont accès aux services de santé maternelle et infantile semblent assumer une part disproportionnée de la stigmatisation. Biais non intentionnelles chez les fournisseurs de soins de santé sont maintenant une frontière critique de la stigmatisation, mais il y a peu d'interventions pour y faire face. Des psychothérapies culturellement sensibles sont nécessaires pour répondre à la détresse psychologique associée à la stigmatisation intériorisée et ces interventions devraient compléter le soutien existant de conseils et de dépistage volontaires. Alors que bénéfique pour prioriser les intérêts des parties prenantes dans les efforts visant à réduire la stigmatisation, la confirmation quantitative de ces positions de consensus sont nécessaires avant le lancement d'interventions spécifiques.

## 1. Introduction

Stigma has been implicated in poor outcomes of human immunodeficiency virus (HIV)/acquired immunodeficiency syndrome (AIDS) care in clinical and community settings (Mukolo, Villegas, Aliyu & Wallston 2013). Stigma reduction is regarded as key to the long-term success of HIV/AIDS prevention and treatment efforts (Pulerwitz, Michaelis, Lippman, Chinaglia & Diaz 2008; Roura, Urassa, Busza, Mbata, Wringe & Zaba 2009; Sengupta, Banks, Jonas, Miles & Smith 2011). Stigma is generally sustained by a complex set of factors that are not easy to address (Alonzo & Reynolds 1995; Corrigan, Watson & Barr 2006; Goffman 1963; Green, Davis, Karshmer, Marsh & Straight 2005; Logie & Gadalla 2009; Mahajan, Sayles, Patel, Remien, Sawires, Ortiz, *et al.* 2008; Major & O'Brien 2005). While it is known that some people are more vulnerable to stigma than others, it is not clear what accounts for variance in vulnerability to stigma in general as well as in specific settings (Mukolo, Heflinger & Wallston 2010). Stigma reduction strategies that work in some contexts (e.g. Western nations) might not work in other contexts, e.g. resource limited, linguistically and culturally diverse regions in sub-Saharan Africa (Mutalemwa, Kisoka, Nyigo, Barongo, Malecela & Kisinza 2008; Nyblade, Stangl, Weiss & Ashburn 2009; Pulerwitz, Michaelis, Weiss, Brown & Mahendra 2010).

Literature describing HIV/AIDS stigma is extensive, but accounts of stigma reduction are few (Brown, Macintyre & Trujillo 2003; Heijnders & Van Der Meij 2006; Sengupta *et al.* 2011). A recent review points to lack of dedicated stigma reduction interventions and good quality efficacy assessment studies (Sengupta *et al.* 2011). Therefore, more descriptions are needed to highlight the diversity and efficacy of stigma reduction interventions that are being tried and tested in relation to HIV/AIDS. While progress has been made to develop theoretic models to assist in the identification and classification of anti-stigma strategies (Heijnders & Van Der Meij 2006; Holzemer, Uys, Makoae, Stewart, Phetlhu, Dlamini, *et al.* 2007; Mahajan *et al.* 2008; Nyblade *et al.* 2009), there is a need to develop and document strategies informed by comprehensive models of stigma, covering dimensions of stigma that tend to be overlooked, such as internalized and institutional stigma (Sengupta *et al.* 2011). As noted by Sengupta *et al.* (2011) there is need for studies and/or interventions whose primary goal is to reduce stigma and for such studies to address issues peculiar to target populations and the context in which those populations experience stigma.

In Mozambique, the need to address the negative consequences of stigma is widely acknowledged and supported among associations of people living with HIV/AIDS (PLWHA), government agencies and nongovernmental organizations (NGOs) involved in HIV/AIDS care. However, to the best of our knowledge, there are no reported systematic (rigorously evaluated and published) studies of HIV/AIDS stigma reduction in Mozambique. Therefore, the domains of stigma that characterize the problem of HIV stigma in Mozambique are not widely reported in the literature and little is known about variance in the manifestation of stigma across socio-geographic contexts, more so between rural and urban settings. Furthermore, interventions to reduce HIV stigma in Mozambique appear limited in scope, most notable are mass media campaigns (TV and bill board advertisements and the use of drama and theatre), the enactment of anti-discrimination legislation by the national government in 2002 and 2009 (UNAIDS 2013), and indirectly, through the scale-up of antiretroviral treatment programs for HIV infected patients (Pearson, Micek, Pfeiffer, Montoya, Matediane, Jonasse, *et al.* 2009). There is also need for a comprehensive theoretic framework to guide the development and critique of context-specific anti-stigma strategies in Mozambique.

We describe one attempt at generating consensus on critical characteristics of HIV stigma and anti-stigma interventions suitable for Zambézia Province, Mozambique, a region that has been impacted by the HIV epidemic and has been targeted for the scale-up of anti-retroviral treatment (ART) since 2006. For example, HIV prevalence in Zambézia Province is estimated at 12.6% among adults 15–49-year-old and 15.3% among women vs. 8.9% among men (INSIDA 2009). This represents a decline in prevalence over time, since in 2004 the adult prevalence for the central region of Mozambique in which Zambézia Province is located was estimated at 20.4% (INSIDA 2009). We describe key dimensions of stigma that a diverse group of stakeholders identified and some of the stigma reduction interventions presumed most suitable for Zambézia Province.

## 2. Methods

### 2.1. Overview

The consensus on stigma was derived through an operations research process undertaken by Friends in Global Health (FGH)-Mozambique and Vanderbilt University (see operations research guidelines by the Population Council at www.popcouncil.org). The goal of this formative research was to develop a comprehensive anti-stigma strategy or bundle of strategies relevant to this setting in Mozambique. We aimed for a strategy that covers an exhaustive range of theorized stigma dimensions and domains, with the view of operationalizing the stigma conceptual framework articulated by Mahajan *et al.* (2008) and the social psychology model propounded by Corrigan *et al.* (2006). In our view, these generic models/frameworks capture a broad range of stigma domains than are reported in stigma reduction studies (Sengupta *et al.* 2011). In addition, we wanted a stigma strategy that is grounded in the lived experiences of a broad spectrum of stakeholders in the HIV/AIDS care in Mozambique and emerges from a consensus-building strategy informed by Delphi principles and deliberative group processes (Green 2008). In our view, this adds rigor to the strategy development process and strengthens the credibility of propositions about stigma and its impacts.

### 2.2. Consensus-building strategy

**Fig. 1.**
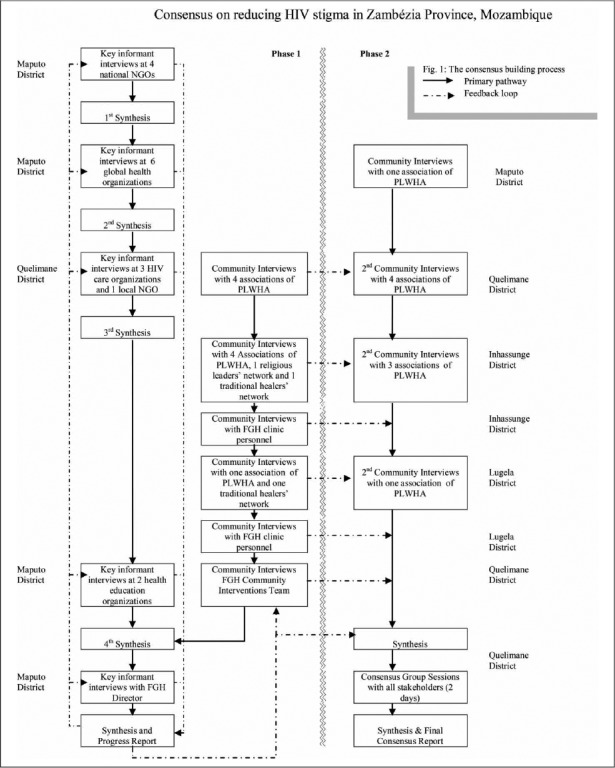
The consensus-building process.

The consensus-building process constituted different levels of consultation, structured in hierarchical format, from initial unstructured interviews with key informants, to more structured workshop sessions on selected topics and potential solutions. Figure 1 details the consensus-building process followed, including data sources consulted, the sequence of data gathering and synthesis followed and the feedback loops adopted. The stakeholders involved at increasingly higher order deliberations included patients’ representatives, healthcare workers and policy-makers. We aimed for a consensus-building strategy that balances the need for the comprehensive coverage of all typical and atypical stigma experiences and interventions on one hand, while generating concurrence among diverse stakeholders on priority stigma issues that need to be addressed and essential stigma reduction strategies on the other hand.

Several qualitative data gathering methods, including 21 indepth interviews with key informants from HIV/AIDS care agencies and institutions, 23 community interviews and 14 hours of consensus group sessions with all stakeholders, were utilized over a 10 months period up to 30 September 2010 (see Table 1). This approach was chosen because numerous healthcare and community development institutions in Mozambique, including individuals and local community-based organizations (CBOs), intervene to address HIV/AIDS stigma, but there is no national-level consensus about stigma reduction strategies or sustained systematic documentation of strategies that have worked and those that have not worked well in various contexts, particularly in Zambézia Province. As noted earlier, there is growing interest to document the changing nature of stigma in Mozambique and the efficacy of interventions to reduce it.

**Table 1.** Primary data sources for the consensus-building process.

Data capture method (and location)	April – May 2010	September 2010	Total
*Key informant interviews (M = 60 minutes)*			
Maputo	16	1	17
Zambézia Province	3	1	4
Total	19	2	21
*Community interviews (M = 80 minutes)*			
Maputo	0	1	1
Quelimane	4	4	8
Lugela (PLWHA)	1	1	2
Lugela (traditional leaders)	1	0	1
Inhansuunge (PLWHA)	3	3	6
Inhansuunge (traditional leaders)	2	0	2
FGH (clinical and community outreach teams)	3	0	3
Total	14	9	23
*Consensus group sessions with all stakeholders (in hours of deliberations)*			
Day 1	0	6	6
Day 2	0	8	8
Total hours of deliberations	0	14	14

### 2.3. Participant selection

Staff from FGH-Mozambique, a designated HIV/AIDS care and treatment partner for the Ministry of Health in Mozambique that has been facilitating the scale-up of HIV services in Zambézia Province since 2007, identified and recruited participants for key informants interviews, community interviews and consensus group sessions based on participants’ knowledge of the history of the HIV epidemic, public responses to the epidemic, and of the day-to-day realities of living with HIV/AIDS in Mozambique and Zambézia Province in particular. Information was gathered only from persons who consented to share their views and experiences. For a chart depicting the scheme followed in carrying out the interviews and meetings, see Fig. 1.

Community interviews were held at eight established and registered associations of PLWHA in Zambézia Province, four in the capital Quelimane, three in Inhassunge District and one association in Lugela District (both districts are predominantly rural). We also conducted community interviews with three networks of traditional leaders (traditional healers and spiritual leaders) actively engaged in district level community responses to the HIV epidemic in Inhassunge and Lugela districts. District selection criteria ensured one district had a high prevalence of HIV infection and the other low, one had a long history of HIV-related public interventions and the other had an emerging scale-up of HIV services. The districts also had other socio-cultural differences that were deemed to reflect the socio-cultural diversity of Zambézia Province that needs to be considered in the design of anti-stigma strategies. We also held community interviews with medical staff (i.e. nurses, pharmacist, laboratory technicians and data managers) at two rural clinics in Lugela and Inhassunge districts and one with the community interventions team of FGH in Quelimane. In total, we conducted 24 community interviews of about 90 minutes each, 15 interviews at first round of consultations and 9 at follow-up (Table 1). Key informant interviews lasted an average of 60 minutes each.

Community interviews were open to all members of the associations or networks, involved persons who are familiar with each other and were conducted in a non-formal (less structured) manner than key informant interviews. Community interviews approximated the manner that each community group naturally talks about HIV and health-related issues. Furthermore, familiarity among group members had the added advantage that opinions and examples of lived experiences were immediately verified (affirmed or challenged) within the group.

The list of key informants, institutions and communities to interview underwent a rigorous vetting process at several FGH-Mozambique staff meetings in Maputo and Zambézia Province to ensure it was comprehensive and representative of known and critical information sources. Since the operational research was part of a program development process (rather than an academic research endeavor), staff responsible for program implementation and the monitoring and evaluation team were motivated to ensure that program development would be informed by data generated from diverse, credible and knowledgeable sources. For completeness, key informants were also asked during the first round of interviews to suggest other knowledgeable persons and institutions that could help inform the investigation.

### 2.4. Interview topic guide

**Fig. 2.**
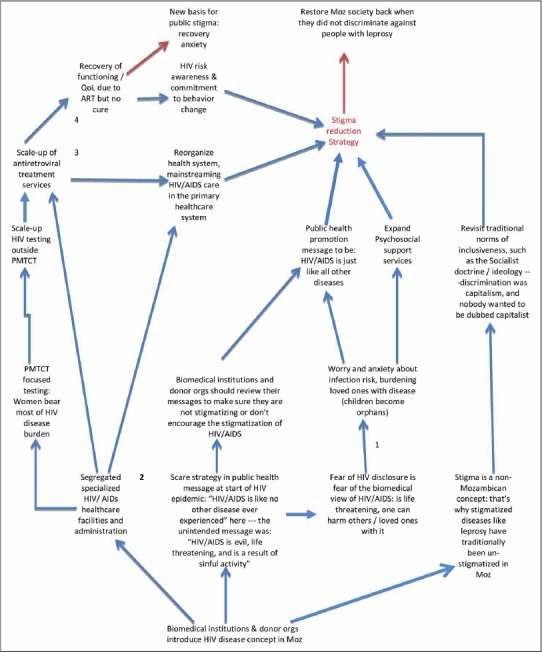
Cognitive map of key interview: SODA goal is to identify ways to reduce HIV stigma in Mozambique.

The interviews were anchored around the following questions: Does HIV/AIDS stigma exist? Has it changed over time? What are the main manifestations of stigma? What are the main consequences of stigma? Which stigma reduction strategies have worked and which have not worked? What are the current stigma reduction needs? The interviews were conducted by two trained interviewers with behavioral/social science backgrounds: one experienced stigma researcher and one with local expertise in HIV care delivery. Both interviewers took detailed notes of the interviews and prompted interviewees for clarifications where necessary depending on the direction of the responses (see Fig. 2). After the first four key informant interviews, interviewers exchanged notes and analyzed them for emerging themes or commonalities and differences in key informants’ views. These emerging themes were then further prompted in subsequent key informant interviews and community interviews, since our ultimate goal was to generate consensus. This iterative and triangulation process was repeated over subsequent interview sessions. Secondly, an interim report was sent to key informants in June 2010 for them to comment on the emerging consensus, allowing them a second chance to contribute to the consensus-building process. In addition, communities interviewed in the first round of consultations were also interviewed in the second round facilitating them to further reflect on the emerging themes. Furthermore, these community groups chose members to represent them at consensus group sessions conducted in September 2010.

### 2.5. Consensus group sessions

Following key informant and community interviews and feedback gathering, a 2-days consensus development workshop was convened, bringing together all the stakeholders engaged in earlier deliberations as well as healthcare policy-makers, clinicians with supervisory responsibilities in health facilities and other professionals whose functions involves interacting with and serving PLWHA in Zambézia Province. The themes for the consensus group sessions were (1) Critical stigma issues to focus anti-stigma strategies based on results of key informant and community interviews and a review of HIV stigma literature, (2) Prioritization of issues, including the types of stigma, and the ranking of issues by the urgency with which they should be addressed if HIV stigma and its consequences are to be mitigated, (3) Intervention concepts – select most appropriate and highly feasible strategies and (4) Capacity needs for strategy delivery (see data analysis below). Consistent with this methodology, the sessions began with presentation of the conceptual framework and results of key informant and community interviews, followed by a plenary session to ensure agreement on validity and reliability (Green 2008). This was followed by a series of small group discussions of themes, each leading to presentation of group propositions and plenary discussions to arrive at a consensus. Day 1 involved all invited participants. Day 2 was attended by program developers and evaluators and by representatives of rural associations of PLWHA that had attended Day 1 deliberations. The goal was to review Day 1 consensus positions based on their relevance to the rural contexts in which stigma reduction interventions are to be delivered as well as their significance and viability. Deliberations also centered on generating consensus on measurable objectives and outcomes of selected interventions as well as the sequence by which interventions should be delivered in order to maximize the likelihood of achieving the anticipated shifts in stigma.

### 2.6. Data analysis

The cognitive mapping technique was used to analyze the first four key informant interviews (see Fig. 2 for an example of one such map), based on Eden and Ackermann's (2004) guidelines for constructing cognitive maps (c.f. Sanderson & Gruen 2011:44–48). Cognitive mapping is a stage in a broader analytic decision-making process called strategic options development and analysis (SODA). SODA is a method for structuring a problem (Sanderson & Gruen 2011:43), in which patterns of thoughts that participants in a decision-making process have on a decision scenario (and are verbally describing in an indepth interview) are graphically captured and represented (see Fig. 2 for an example of a cognitive map). SODA is most useful when the following conditions exist: (1) There are many stakeholders/participants in the decision process; (2) There are many desirable but not yet clearly articulated outcomes, each with varying degrees of feasibility and probability of occurrence; (3) There is need to articulate the paths to certain outcomes or select the most desirable and agreeable path to a given outcome; and (4) Final decision-makers are cognizant of the fact that achieving the desired outcome or implementing the outcome-generating activities requires commitment of other stakeholders. Not all participants in the SODA process will be conscious of their own cognitive map *a priori* and each participant's cognitive map might become apparent as the competent analyst elicits participants’ views, usually during an indepth one-on-one interview. Ideally, the analyst develops a meta-map from individual cognitive maps to represent emerging consensus about the outcome(s) and strategic options for reaching the outcome(s). The consensus group then works from this meta-map to make final decisions based on, among others, feasibility of each option and probability of achieving the outcome following each optional strategy/path.

Our consensus-building process was planned so that, once a clear map was evident from a primary set of indepth interviews, each subsequent level of consultation would try to capture distinctively new insight and progressively lead to a distilled list/description of issues, narrowing down the list of desirable and testable decision options (see results). Rather than construct a meta-map, due to time constraints, phase 1 cognitive mapping helped focus the consensus-building workshop sessions on high order goals and means for reaching them (Sanderson & Gruen 2011:46–47). In consensus-building workshops, we explored actions for each option to describe their potential as intervention points. New options or tails to existing concepts and options were also identified (Sanderson & Gruen 2011). The process worked by negotiation among participants, each option being reviewed for its merits and each proposer having to logically support her/his choice, so merits of various views and options were estimated and compared. The group had to agree on criteria for choosing among alternative goals and ways of achieving them. This enabled experience-gathering from many diverse stakeholders and the harmonization and integration of these experiences and learning into alternatives for action.

### 2.7. The conceptual framework operationalized

We utilized the labeling theory informed conceptual framework described by Mahajan *et al.* (2008) and reiterated by Earnshaw and Chaudoir (2009) because it captures a broad range of stigma domains than are reported in most HIV/AIDS stigma studies. Support for the frameworks also came from the cognitive maps of the first four key informant interviews (see Fig. 2 for an example).

Stigma is a multifaceted multidimensional construct (Goffman 1963; Link & Phelan 2001; Mahajan *et al.* 2008). Labeling theory posits that stigmatization is a sequential process that begins with labeling (based on perceived deviance from a given norm) and negative stereotyping of the deviant entity by others, which leads to separation and status loss (or devaluation) of the labeled entity and subsequently discrimination (Link & Phelan 2001; Mahajan *et al.* 2008). HIV/AIDS literature details damaging labels and stereotypes of PLWHA (Earnshaw & Chaudoir 2009; Uys, Chirwa, Dlamini, Greeff, Kohi, Holzemer, *et al.* 2005), some of which are often transferred or projected onto those associated with PLWHA such as family members, friends and healthcare providers (Surkan, Mukherjee, Williams, Eustache, Louis, Jean-Paul, *et al.* 2010). These interpersonal and public aspects of stigma can be internalized by some individuals through socio-psychological processes of stereotype awareness, acceptance and concurrence (Corrigan *et al.* 2006), leading to potentially profound cognitive and behavioral consequences such as self-esteem decrement, psychological distress and maladaptive coping. To our knowledge, models and theoretical frameworks of HIV stigma have not fully incorporated these aspects of stigma, as has been done in relation to mental illness stigma (Corrigan *et al.* 2006; Yang, Kleinman, Link, Phelan, Leed & Good 2007). Sengupta *et al.* (2011) reviewed results of 19 published stigma reduction studies and found that none assessed internalized (i.e. self) stigma. Corrigan *et al.*'s self-stigma model (Corrigan *et al.* 2006; Watson, Corrigan, Larson & Sells 2007; Yang *et al.* 2007) is particularly appealing, from an intervention design perspective, because it recognizes the importance of belonging to social networks or peer support groups that consider stigma illegitimate and actively advocate for legal and social reform. The self-stigma framework therefore, provides theoretic basis for designing targeted context-specific interventions to build up positive psychological resources (including coping skills) that individuals can utilize to deal with the public stigma of HIV/AIDS and mitigate self-stigmatization and its negative consequences. We also considered other personal factors that have been theorized to account for variance in self-stigmatization based on the conceptual framework proposed by Major and O'Brien (2005), such as the significance of identity threat and sensitivity to social rejection (Steele & Aronson 1995). These psychosocial constructs help capture the dynamics of living with HIV/AIDS in a largely hostile social milieu, but have not been considered in resource limited settings (Mukolo *et al.* 2013).

Our operational framework also ensured that stigma issues are viewed within the backdrop of the stigma trajectory proposed by Alonzo and Reynolds (1995), which helps to account for the effect of changes in the bio-physiology of HIV/AIDS – from (phase 1) the time when one is at risk of HIV infection but worries about consequences of infection, through (2) the HIV diagnosis when one comes to terms with his/her new/changed identity, (3) the time when one is living between illness and health, up to (4) the manifest phase when one experiences AIDS and the likely passage to social and physical death (Alonzo & Reynolds 1995). The stigma trajectory was particularly appealing to us because it is grounded in the natural history of HIV infection and helps one consider how stigma might be influenced by the history of and public responses to the epidemic in Zambézia Province. Therefore, although the consensus-building process was being undertaken during one of many phases in the Mozambican HIV epidemic, the operational framework allowed for both retrospective and prospective reflections. By doing so, we believe that we partially compensated for the absence of longitudinal studies of changes in public attitudes and behavior.

## 3. Results and discussion

Outputs have been synthesized into a logical framework and are being turned into an implementation plan. The following eight consensus positions were captured.

*Consensus one*: Stigma has declined over time in tandem with and corresponding to the history of the HIV/AIDs epidemic and of the scale-up of public health interventions in Zambézia Province, Mozambique.

The key changes have been in the toning down of negative public attitudes toward the HIV infected – more precisely consensus is that the uninfected are less inclined to enact their stigmatizing attitudes and behaviors and that tolerance for PLWHA has increased in the general population. Key drivers of reductions in the levels of stigma have been (a) acquisition of knowledge that most known modes of HIV transmission can be managed/controlled to prevent HIV infection, boosting confidence that risk of contagion can be minimized and enhancing prevention self-efficacy and, (b) knowledge that efficacious therapies exist and thus the HIV infected (even those with advanced HIV disease) need not die from infection or experience poor quality of life. The stigma of HIV infection/disease is mitigated when the dominant (socially held) image and prognosis of HIV disease changes from life-threatening to chronic disease and through decrement in fear and anxiety about HIV infection among both the infected and uninfected. For detailed description of the link between the HIV stigma and the natural history of HIV disease, see Alonzo and Reynolds (1995) and Brown *et al.* (2003). The change is attributed to national level interventions to reduce stigma, notably legal reforms and informational stigma reduction strategies (i.e. mass media-delivered public education campaigns). As has been noted in relation to other anti-stigma initiatives (Brown *et al.* 2003), we do not know how deep or superficial these perceived changes have been and which domains of stigma have been impacted the most. Therefore, we need properly designed studies that are dedicated to tracking measurable changes in public stigma over time in Zambézia Province.

The scale-up of efficacious anti-retroviral therapies, to the extent that it changes the image of HIV disease from a life threatening condition (extremely stigmatizing due to existential threat) to a chronic illness (less stigmatizing and induces hope due to the probability of clinical remission and enhanced quality of life) interferes with the stigma trajectory. Hence, we considered the scale-up of combination ART (cART) as a clinical or biomedical stigma reduction strategy, particularly in high HIV prevalence contexts like Zambézia Province. The strategy might be particularly effective where there are highly visible treatment experienced patients who have experienced dramatic clinical remission following cART commencement.

Nonetheless, we were cognizant that stigma reduction might not occur in a linear fashion. For example, it has been noted in some contexts that cART efficacy can have both abating and incremental effects on HIV stigma (Roura *et al.* 2009). Roura *et al.* (2009) observed that improvements in the health and functioning of persons on long-term cART engenders positive attitudes toward HIV disease among the non-infected, but does not change dominant (lay) attributions about HIV transmission and the belief that the HIV infected are fundamentally flawed and incapable of changing their risky and socially deviant behaviors. cART efficacy likely makes HIV infection invisible (i.e. purges externalized bodily characteristics by which to tag the infected), making it challenging to apply lay criteria for screening the HIV infected and exerting social control over them. As noted by Roura *et al.* (2009), cART efficacy might create new bases for social anxiety about HIV infection in some settings. In Fig. 2, a similar outcome is captured in the cognitive map of one of the key interviewees.

*Consensus two*: Levels of perceived stigma are likely to be greater than enacted stigma because of the intensity of public stigma enacted in earlier phases of the epidemic and the gravity of the consequences of stigma experienced in these earlier times.

This is partially indicated by a near pervasive fear of sero-status disclosure and considerable investments in the management of information about one's sero-status and use of HIV-related services (Alonzo & Reynolds 1995). The discrepancy might also reflect the presence of internalized stigma. As noted in the methods section, internalized can lead to profoundly negative cognitive and behavioral consequences such as self-esteem decrement, psychological distress and maladaptive coping. Therefore, residual fear of public stigma is likely to mask real positive changes in public attitudes and behavior toward PLWHA in Zambézia Province. Such deep-seated fear (Brown *et al.* 2003) might also indicate the prevalence of maladaptive coping with public stigma among some PLWHA or those who worry about HIV infection risks. There is need, therefore, to investigate this assertion because, if true, one might expect generational disparities in the discrepancy between perceived and enacted stigma – with lesser discrepancy and greater willingness to utilize HIV services among younger than older persons in Zambézia Province. The underlying psychological impact of public stigma might require that counseling and support strategies for reducing stigma be implemented in combination with informational strategies (Heijnders & Van Der Meij 2006).

*Consensus three*: As a direct product of observations in consensus one and two, social withdrawal and secrecy (theoretically consistent consequences of public/community stigma) might be maladaptive responses to public stigma, since public stigma has declined over time in Zambézia Province.

The persistence of such responses indicates potential internalization of public stigma by PLWHA. According to the self-stigma model (Corrigan *et al.* 2006), victims of public stigma might differ in their responses to stigma by the extent to which they consider public stigma to be legitimate. Variance in perceived legitimacy primarily reflects differences in the tendency to concur with negative stereotypes widely held in one's social milieu. According to this theory, those who consider public stigma to be illegitimate tend to be represented among persons who reject negative social stereotypes and respond to the public stigma with righteous anger – via protests, litigation and other forms of communicating disapproval and seeking redress for damages (Corrigan *et al.* 2006). Therefore, maladaptive coping with stigma in this context is likely among PLWHA who concur with negative public stereotypes. Internalized stigma might be indicated by the elevated levels of psychological distress often observed in samples of HIV infected persons, either pre-and post-HIV testing or among the treatment experienced (Arendt 2006; Atkinson, Higgins, Vigil, Dubrow, Remien, Steward, *et al.* 2009; Berger, Schad, von Wyl, Ehlert, Zellweger, Furrer, *et al.* 2008; Burack, Barrett, Stall, Chesney, Ekstrand & Coates 1993; Eller, Corless, Bunch, Kemppainen, Holzemer, Nokes, *et al.* 2005; Gore-Felton & Koopman 2008; Ickovics, Milan, Boland, Schoenbaum, Schuman & Vlahov 2006; Kaharuza, Bunnell, Moss, Purcell, Bikaako-Kajura, Wamai, *et al.* 2006; Leserman 2008; Owe-Larsson, Sall, Salamon & Allgulander 2009). Therefore, screening for psychosocial distress might help identify PLWHA who are at risk of internalizing public stigma in Zambézia Province. Psycho-educational anti-stigma strategies (Brown *et al.* 2003; Sengupta *et al.* 2011) should be focused on those at risk of psychosocial distress, helping them acquire knowledge of alternative (externalized) responses to public stigma (such as advocacy/protest, strategic sero-status disclosure and help seeking). In Mozambique, PLWHA have the opportunity to belong to voluntary associations of PLWHA and healthcare-provider-initiated peer-support groups. Some of these social networks have a strong anti-stigma agenda while others are focused on non-stigma aspects of HIV/AIDS care such as ensuring fidelity to care and ART through improvements in logistics and treatment self-efficacy. Some local support groups are coupled to complex social networks that have far-reaching social and legal reform agendas pursued at multiple geo-political levels. These social networks provide potential avenues for developing theory-driven, targeted and context-specific interventions that help build up positive psychological resources (including coping skills), mitigating self-stigmatization and its negative consequences. Obtaining the support of popular opinion leaders in local communities would be critical to the success of such interventions.

*Consensus four*: Male participation in maternal and child health programs, coupled with stigma focused educational interventions, are likely to reduce the disparity in knowledge of sero-status and women's vulnerability to stigma.

Despite consensus one, two and three above, enacted stigma still exists in some segments of Zambézia Province, as evidenced by participants’ recall of recent (publicized) instances of physical and verbal abuse and neglect of persons with advanced disease that culminated in acute hospitalization and, in some cases, suicide. We have not been able to confirm the suicide claims. However, the consensus is that the burden of enacted stigma is disproportionately skewed toward women of reproductive age as they are more likely than men to be tested for HIV infection, e.g. via maternal and child health interventions to prevent mother-to-child transmission (PMTCT) of HIV that are being scaled-up by the Ministry of Health in the province. As advocated elsewhere in the Southern African region (de Paoli, Manongi & Klepp 2004), increasing male involvement in PMTCT programs is likely to moderate the degree of stigma enacted toward women in the province.

*Consensus five*: Notwithstanding consented national-level legal and social norm reforms, institutional stigma, as reflected by negative attitudes and behaviors of healthcare workers and discriminatory policies and practices of healthcare institutions, persists. Institutional stigma is likely the most significant form of HIV stigma in Zambézia Province and is probably the single most significant psychosocial barrier to the utilization of HIV services.

Institutional stigma is one of four types of stigma highlighted, based on deliberations about who is being stigmatized and the source (or context) of stigma – the other three being public stigma, courtesy/associative stigma and self-stigma (Andrewin & Chien 2008; Mukolo *et al.* 2010). Institutional stigma was further subdivided into professionals’ attitudes and behaviors and organization policies and operational procedures. The consensus was that, at the level of healthcare professionals, institutional stigma cannot be explained solely by lack of factual knowledge about HIV transmission, prevention and treatment, but more likely by unintended biases in healthcare professionals’ beliefs and behaviors toward HIV infected patients and/or inadvertent consequences of implementing current care delivery practices. The assertion is partially supported by the fact that no targeted systematic stigma reduction interventions have been developed for healthcare settings in Zambézia Province. Elsewhere it has been noted that, while there is an increasing willingness to treat HIV infected patients, factual knowledge of HIV transmission routes and poor infection control practices might inadvertently increase the fear of contagion among health workers (Brown *et al.* 2003). Hence, interventions are needed among healthcare professionals to improve stigma awareness and enhance the quality of provider-patient interaction.

*Consensus six*: Stigmatizing and discriminatory standard operating procedures (SOPs) in healthcare institutions are a barrier to the uptake of HIV testing, care and treatment services.

Many participants, particularly those enrolled in care narrated specific instances of enacted stigma or stigma triggers they experience in the process of accessing and utilizing existing care. These had to do with policies and standard operational procedures implemented at healthcare facilities. Most representatives of the healthcare providers that participated in the consensus-building exercise seemed unaware of these institutional aspects of stigma. In addition, systems to facilitate patient input in healthcare quality improvement were poorly developed and most patients ascribe to a culture of deference to authorities, particularly in rural settings. Similar observations have been made in other countries (Mahendra, Gilborn, Bharat, Mudoi, Gupta, George, *et al.* 2007; Nyblade *et al.* 2009). Nyblade *et al.* (2009) reviewed studies aimed at mitigating stigma in healthcare facilities and noted provider unawareness of what stigma looks like as well as lack of effective ethics/norm enforcement mechanisms needed to deviantize (Schur 1983)^1^ stigma enacted toward patients.

While acknowledging that treatment efficacy has a positive impact on public attitudes and behaviors, study participants considered current guidelines on cART commencement and HIV care as favoring persons with advanced HIV disease, which inadvertently perpetuates the negative image of HIV disease, i.e. most people in Zambézia Province only know about advanced forms of HIV disease. Policy shifts toward earlier cART commencement and pre-ART care that emphasize health maintenance at early stages of HIV disease, would reinforce the positive image of HIV disease that emanates from treatment efficacy. The public health significance of such policy changes might be an increase in the number of HIV infected persons who present early for HIV diagnosis.

The consensus was that reducing institutional stigma requires a partnership between healthcare providers and HIV infected patients because, on one hand, patients are better placed to note unintentional bias in SOPs and personal attitudes and behaviors that are oblivious to providers, while providers can facilitate patients to take a more active role in ensuring the success of clinical/bio-medical approaches to reducing HIV stigma. Hence, the consensus was to adopt a partners-in-health approach to address institutional stigma in healthcare settings.

*Consensus seven*: Lack of adequate information about HIV, stigma and the legal rights and entitlements of PLWHA are still the main drivers HIV/AIDS stigma in all settings (public/community, self and institutional) in Zambézia Province.

This might not be unique to Zambézia Province or Mozambique as similar observations have been made in some regions of neighboring South Africa (Campbell, Nair, Maimane & Nicholson 2007). Under consensus one, reductions in externalized forms of public stigma at the national level are attributed to better knowledge about HIV transmission routes and preventions. However, emerging findings from a province-wide survey of health-related attitudes and behaviors indicates that most adults in Zambézia Province have limited knowledge about HIV transmission and prevention (Vergara, Blevins, Vaz, Manders, Calvo, Arregui, *et al.* 2010). As many as 70% of those surveyed in the province could not provide one correct mode of HIV transmission or stated they did not know how HIV is transmitted (Vergara *et al.* 2010). A link was also reported between willingness to negatively label and socially exclude PLWHA and low levels of HIV transmission knowledge in data from the same survey (Mukolo, Blevins, Victor, Vaz, Sidat & Vergara 2013). Although further investigations of these observations and assertions are needed, public health education interventions to improve knowledge of facts about HIV transmission and prevention might still be the way to reduce HIV stigma in this community. Mukolo *et al.* (2013) also reported low levels of willingness to stigmatize PLWHA among female heads of households who were confident that the legal system would adequately protect them if they needed protection. There is a need to increase awareness of the anti-discrimination legislation that was enacted by the national government in 2002 and 2009, particularly in rural provinces and districts. Implementation of these legal provisions is likely to increase public confidence in the legal protections that are now available to PLWHA. Follow-up operational research should track the impact of improvements in the knowledge of HIV transmission/prevention and the legal rights of PLWHA in the four domains of stigma highlighted in this consensus-building process – public, courtesy, institutional and self-stigma.

*Consensus eight*: Stigma reduction approaches likely to work include (1) public education (informational strategies) to address under and misinformation about HIV transmission, prevention and treatment as well as lack of knowledge about the nature of stigma and how it occurs, (2) skills-building to provide positive coping skills among those susceptible to negative consequences of public stigma, (3) dissemination of information about and/or enforcement of laws, policies and procedures that protect the stigmatized and punish stigmatizers particularly among service providers, (4) role modeling by community leaders (traditional, political & religious) to promote social norm reform and enforcement (breaking silence, denial, fear and institutionalized violence) and (5) protest by PLWHA and their associates against stigma enacted in community settings and healthcare institutions. These strategies have been reported in Southern Africa and elsewhere (Pulerwitz *et al.* 2010; Sengupta *et al.* 2011). Follow-up operational research studies should evaluate the comparative efficacy of these stigma reduction strategies, particularly in rural settings.

## 4. Limitations

Despite these key observations, the consensus is not representative of all NGO/CBOs and associations in the Maputo and Zambézia Province, nor is it representative of all PLWHA in the Maputo and Zambézia Province. We consulted with those PLWHA who have joined established associations/organizations and were willing to share their views and opinion at public forums. There was also less than optimal female participation in the group discussions we facilitated and most of the key informants we consulted were male. Even in the group meetings that were dominated by women, we had to ensure that women's voices were heard because the few males in attendance tended to dominate proceedings. However, when directly asked to contribute, most of the women in attendance had no problems articulating their views and opinions.

We have also relied on qualitative and exploratory methodology for building consensus. The approach was deemed appropriate because our primary goal was to identify meta-themes in a rapid appraisal fashion. We did not prioritize individuals’ opinions but institutional and community level experiences with stigma as perceived by informants who were knowledgeable about the institutional and community dimensions of HIV stigma. The themes and hypothesized relationships and patterns outlined, therefore, need to be tested (and hopefully confirmed) via more rigorous and systematic (quantitative) studies.

However, the main strength is that this is one of few in-depth inquiries into HIV/AIDS stigma in Zambézia Province. We also assembled and evaluated views from a broad cross-section of the community of interest, including service-users and PLWHA, clinicians and others involved in the provision of services, policy-makers, advocates, governmental and nongovernmental institutions and civil society organizations, traditional healers and religious leaders and men and women from urban and rural locales. All the national organizations and association of PLWHA we consulted acknowledged the need for a nationwide systematic study of stigma and its consequences as well as documentation of effective stigma reduction strategies and approaches. We hope that the themes and stigma-reduction strategies we highlight provide the backdrop and rationale for nationwide assessment of HIV stigma in Mozambique.

## 5. Conclusion

To our knowledge, this was a first attempt to develop consensus about the nature of HIV stigma and ways to reduce it in Zambézia Province, Mozambique. Even though some of these theoretic frameworks and concepts, e.g. self-stigma model (Corrigan *et al.* 2006) or the identity threat construct described by Major and O'Brien (2005) in respect to stigma, have not been directly applied to HIV or studies of stigma in Mozambique, they have been found to be applicable across diverse cultures and countries. For example, Cheng-Fang, Cheng-Chun, Yu, Tze-Chun, Ju-Yu and Chih-Hung (2005) applied the self-stigma model among depressed people in Taiwan, and Angermeyer *et al.* (Angermeyer & Dietrich 2006; Angermeyer & Matschinger 2003) have tested the model in Germany. Yang *et al.* (2007) have adapted the model for testing among samples in China or Americans of Chinese cultural affiliation. We also borrowed Brown *et al.*‘s (2003) classification of stigma reduction strategies – informational, skill-building, counseling/support and contact with affected groups (Brown *et al.* 2003; Sengupta *et al.* 2011). Since the latter was not useful for describing strategies to reduce institutional stigma (which involves changes in national legislation and organization policies and procedures), we also utilized strategy classification taxonomies used in organization behavior/analysis literature (Chaffee 1985; Chrisman, Hofer & Boulton 1988).

The overriding viewpoint from a broad spectrum of reviewers is that responses to the HIV/AIDS epidemic have had a positive effect on enacted forms of stigma, particularly at community levels. However, there is likely excessive residual fear of HIV disease and community attitudes that some individuals (i.e. the already infected and worried well) struggle with. The lingering concern about public stigma reflects the strength of stigma enacted in earlier phases of the epidemic. This internalization of public stigma (and subsequent psychological distress) likely restricts access and utilization of available health care, particularly among the less targeted subsets of the rural population (i.e. those of higher socio-economic status and men). Given strong evidence indicating the negative impacts of psychological distress on HIV disease progression among the treatment experienced (Chida & Vedhara 2009; Mukolo & Wallston 2012), addressing the psychological implications of internalized stigma should be prioritized among HIV care and treatment programs.

Pregnant women testing positive through maternal and child health services (like PMTCT) are vulnerable to enacted stigma and deleterious consequences such as divorce/separation and withdrawal of essential social support. The positive externalities of current pre- and post-test counseling seem less protective to those subgroups of men and women who struggle with stigma. Given residual fear of public stigma and the likely enactment of stigma post-voluntary counseling and testing (VCT), it might be beneficial to have culturally sensitive interventions dedicated to addressing psychosocial distress post-VCT. Interventions to increase men's support and participation in maternal and child health services would also help reduce women's vulnerability to stigma.

In addition, our consensus population, which included persons who use public HIV/AIDS care services, singled out unintentional biases among healthcare providers (individuals and institutions) as critical psychosocial barriers to current healthcare access and utilization. However, there are few (if any) interventions that are specifically designed to address stigma in healthcare settings in Zambézia Province and elsewhere (Nyblade *et al.* 2009; Sengupta *et al.* 2011). The consensus is that stigma reduction strategies in Zambézia Province should be led by associations of people living with HIV working in partnership with healthcare providers. Associations of PLWHA need to be resourced to acquire requisite capacity to partner with providers to improve the quality of HIV care. Providers need to be supported to effectively address the unintended consequences of standard operational procedures and deep seated biases that likely compromise patients’ access and use of services. This likely entails a paradigm shift in the patient-provider relationship that is likely, also, to generate positive externalities for the broader system of care. The culture change requires support by policy-makers and healthcare providers and the change process needs to be strategically managed.

It would be advantageous if similar stigma audits were to be conducted in other provinces, to tease out the extent to which our findings are mirrored nationwide. At the time of this study, a number of institutions, supported by UNAIDS-Mozambique, were preparing to undertake a national study of HIV stigma based on the Stigma Index compilation methodology (IPPF & UNAIDS/WHO 2010).

## Note

1. The term ‘deviantize’ is used here in the tradition of Schur (1983), meaning to officially designate certain behavior socially unacceptable, behavior that would otherwise be socially acceptable, go unnoticed or be considered unproblematic. In this case, the implication is that the behaviors and attitudes in healthcare settings are not inherently deviant unless socially constructed as such. Anti-stigma interventions will, in essence, be successful in the Zambézia Province to the extent that they help reconstruct particular provider behaviors and practices as morally offensive.
